# Synergies between Small RNA and Protein Biomarkers for Early Detection of Cognitive Impairment – results from EPAD

**DOI:** 10.1002/alz70856_099297

**Published:** 2025-12-24

**Authors:** Tobias Sikosek, Marco Heuvelman, Jagoda Mika, Mustafa Kahraman, Julia Jehn, Maurice Frank, Alberto Daniel‐Moreno, Jessika Ceiler, Jasmin Skottke, Marta Sanchez‐Delgado, Patrick Neubert, Christina Rudolf, Kaja Tikk, Rastislav Horos, Jeffrey L. Cummings, Josie Butchart, Craig Ritchie, Jean Manson, Bruno R Steinkraus

**Affiliations:** ^1^ Hummingbird Diagnostics GmbH, Heidelberg, Germany; ^2^ University of Nevada, Las Vegas, Las Vegas, NV, USA; ^3^ Chambers‐Grundy Center for Transformative Neuroscience, Las Vegas, NV, USA; ^4^ Centre for Clinical Brain Sciences, Edinburgh, United Kingdom; ^5^ Scottish Brain Sciences, Edinburgh, Scotland, United Kingdom; ^6^ University of St Andrews, St Andrews, Scotland, United Kingdom

## Abstract

**Background:**

Alzheimer's disease (AD), the most common form of dementia, is typically diagnosed using the AT(N) framework, which assesses amyloid, tau, and neurodegeneration biomarkers. However, this framework has limitations, particularly in predicting cognitive decline among amyloid‐positive individuals.

**Method:**

This study explores the synergistic potential of combining small RNA biomarkers with established protein markers to improve the early detection of AD. Using data from 1,913 participants aged 50+ from the European Prevention of Alzheimer's Dementia (EPAD) clinical trial, all free of dementia at enrollment, we performed ultra‐deep small RNA sequencing on whole blood samples. A refined sequencing protocol reduced erythroid RNA interference, enabling the identification of rare biomarker RNAs. We define high and low amyloid groups based on a cutoff on the *p*‐tau_181_/Ab_1‐42_ ratio as determined from cerebrospinal fluid.

**Result:**

Analysis revealed small RNAs that predicted early cognitive decline (Clinical Dementia Rating of 0.5) with an AUC of ∼0.7, which increased to 0.77 within the high‐amyloid subgroup. Functional evaluation linked these RNAs to dementia‐relevant pathways, including neuronal, cardiovascular, and inflammatory activities.

**Conclusion:**

Our findings highlight that combining small RNA and amyloid protein markers significantly outperforms either biomarker type alone, offering a more robust and precise approach to early AD detection. This integrated strategy addresses current gaps in the AT(N) framework, enhancing stratification and enabling more effective interventions. Small nucleolar RNAs and microRNAs emerge as promising candidates for further exploration as part of this synergistic diagnostic model.

